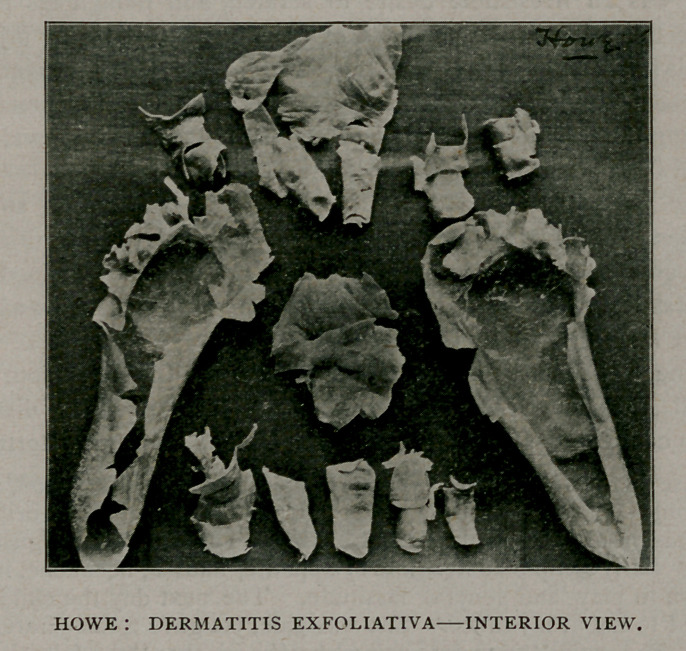# Dermatitis Exfoliativa

**Published:** 1901-02

**Authors:** William A. Howe

**Affiliations:** Phelps, N. Y.


### DERMATITIS EXFOLIATIVA.

By WILLIAM A. HOWE, M. D., Phelps, N. Y.

THE subject which I have chosen to present for your consideration on this occasion, is one which belongs to the domain of the dermatologist rather than to that of the general practitioner. The highly interesting experience which has fallen to my lot with this disease, furnishes my only apology for entering this field of special work in which I am so consciously inefficient. That the task is no easy one is fully appreciated; that others may do it far better justice is freely admitted; that your liberal criticism may prove conducive of some good, is hopefully anticipated.

To me, as I dare say to most of you, this disease has been of rare occurrence. In only three instances, during the last twelve years, has it come under my observation, and then, strange as it may seem to some of you, not until several months after one patient had died from it, and five years after the first case was seen, was I able to make a positive diagnosis.

Two of the cases appeared in the same individual, four years apart; the third one in a child three years of age. In all three of them, the cutaneous and constitutional symptoms were very severe in character and persistent in duration. That of the child resulted in death, the other two in recovery.

Those having a favorable issue so closely resembled each other in every particular that to attempt to describe them separately would only result in a needless repetition. This paper, therefore, will contain a review of only one of the two attacks from which the patient suffered.

My decision to report these cases has been influenced by several considerations. Foremost among them is a desire to increase the number of such cases recorded, hoping, in a measure, to encourage a more exhaustive study of the subject, and a more comprehensive knowledge of its real nature. Its rarity should certainly offer no excuse for its neglect. On the contrary, it should stimulate on our part a still greater determination to search out every possible case, and study it in detail.

Strange as it may seem, many of our standard text-books on dermatology are sadly deficient in their treatment of this interesting disease. Some of them devote practically no space to its considera-

1. Read at the thirty-third annual meeting of the Medical Association of Central New York, held at Rochester, N. Y., October 16, 1900.

tion. Others, though more liberal in their treatment of the subject, still fail to furnish the detailed description which one might reasonably expect to belong to a disease capable of assuming such severity. Though comparatively rare to the dermatologist, it is particularly so to the general practitioner. To such of us as follow our vocation in the rural districts, far fewer opportunities are offered for observing rare forms of disease, and greater difficulty is often experienced in correctly diagnosticating them. For that reason the country practitioner, more than all others, is dependent upon his library to assist him to properly diagnosticated these obscure, perplexing cases.

Though the writer has within his library three standard text-books devoted exclusively to venereal and cutaneous diseases, none of them enabled him to arrive at a positive diagnostic opinion when the disease first presented itself for solution. I admitted candidly to the family that I had never before seen anything exactly like it; that the records at my disposal did not give its parallel, and that, further than its being some violent form of dermatitis, I could not say. Fortunately, for my retention in the case, this seemed to satisfy the family, and I continued in its management. Inasmuch as it pointed to a favorable end or recovery, the family did not deem it necessary to call another physician in consultation. In the second case, however, two consultants were called. Both were general practitioners, of many years of wide experience. To them, as to me, the disease was baffling. Our united efforts availed nothing, the patient succumbing after six weeks of almost indescribable suffering. It was not until my third case was well on its road to convalescence, that I succeeded in obtaining a correct diagnosis of this strange malady. For this valuable information I am indebted to Dr. E. Wood Ruggles, of Rochester, whose familiarity with the disease enabled him to quickly recognise it.

Not only were these cases extreme in their severity, but of a shorter and more defined course than is generally expected in this disease. Many other cases are attended by far less disturbances than were those which furnish the data for my report.

Do we recognise these milder ones as such, or do we, as might easily be done, include them in some of the exanthemata? I am not prepared to state positively whether I have ever seen more than these three cases. It is more than probable that I have, but failed to recognise their real nature, and thereby wrongly diagnosticated them.

Most of you, like myself, have undoubtedly seen eruptive diseases, in which you were never fully satisfied as to their positive diagnosis.

Some you may perhaps have called a case measles, some scarlatina, some erythema, and yet down in your own conscience a grave doubt existed as to just what you were dealing with. The fact that in many of these cases frequent exposures were given to children with no contagion only added doubt to doubt in your already unsettled mind.

Might it not be possible that some of these noncontagious cases should be found to be a mild form of dermatitis exfoliativa? Should we not be more vigilant in watching for this disease, and endeavor more frequently to differentiate it from those conditions for which it might easily be mistaken?

Cases I. and II.—H.F.B., male, aged 44 years, married. Father died at 71, with diabetis mellitus; one brother at 24, and one sister at 43, with the same disease. Mother died at 70 with dilatation of heart. Except these three cases of diabetis mellitus, his family history is good. The subject of this report is a farmer by occupation and always has been. He is of exemplary habits, naturally strong and active, and except the two attacks, which furnish a portion of the data for this report, he has never experienced any serious illness. He was prostrated by the first in March, 1895, and by the second in December, 1899, and January, 1900. During the fall of 1899, or just preceding his last attack, he complained of unnatural dryness of the skin, with more or less itching and burning. This was particularly noticeable on the hands and feet. No constitutional symptoms were present during this early prodromic period. These first manifested themselves on December 20, 1899, at which time he complained of slight vertigo on rising in the morning, headache, anorexia, indisposition to work, more or less exhaustion, chilly feelings, aches and pains in back and extremities, increased dryness of the skin, great itching and burning, and an apprehension of some impending sickness of a severe nature.

These premonitory symptoms rapidly grew worse and others were soon added. Within forty-eight hours of the advent of the early symptoms, the man was profoundly sick. He was now completely prostrated in bed. The itching and burning of the skin now involved the entire body, as did also a deep erysipelatous eruption. These were very severe and pronounced in their character. The face was so swollen and edematous, that it was with difficulty that he could see out of either eye. His facial features were so distorted that his most intimate friends would scarcely have recognised him. He was delirious, muttering incoherently, picking at the bedding and extremely restless.

Though now covered with a severe dermatitis and exhibiting such severe constitutional symptoms, his temperature registered only 101° and his pulse was normal. In fact, throughout the entire disease his pulse did not rise much beyond 72 and 102 2·5 was the highest point reached on the temperature chart. This was on the third day of the disease, by which time his hands and feet were so badly swollen as to make it almost impossible to move them. This intense inflammation continued with no abatement until December 25th, at which time occurred a slight amelioration of its severity.

Coincident with this seeming improvement, came an increase in the swelling, which produced almost intolerable suffering, So great were the pain and itching which attended this condition that opiates were necessarily given freely to relieve these distressing symptoms. The next day, the deeply reddened skin began to present a dried, dead appearance and crack open in many places. This evidently arose from the destructive influence of the intense inflammation on the vitality of the skin, together with the distension accompanying it.

HOWE: DERMATITIS EXFOLIATIVA—EXTERIOR VIEW.

It was at this time, or on the sixth day of the disease, that the first exfoliation occurred. This was at first slight, but like the other symptoms, rapidly increased until the man was actually skinned alive. Indeed, in certain portions of his body, a secondary exfoliation occurred, which added not a little to his intense suffering. By December 27th, extensive surfaces were exfoliating. On this day the entire palm of the thumb came off en masse. The character of the exfoliated skin varied materially in different parts of the body. In some places, especially on the face, it would be fine and bran like; in others, as on the trunk, it would roll up in great sheets, while in others as on the hands and feet, it would be thrown off in a perfect cast.

This last feature of the case has been of particular interest to me. The specimens obtained have been preserved and are exhibited for your inspection; also, a photograph of their exterior and interior surfaces, taken soon after their removal. They were all exfoliated between the seventh and fourteenth days of the disease. Some of the fingers you will notice were stripped off, much as you would turn the finger of a glove inside out. Those of the soles of the feet were removed by making an incision along the superior surface of each toe and slipping off the epidermis, as you see it in the specimens. You will also notice that they are still, more than nine months since their removal, in a state of excellent preservation. Nothing has been applied to them in the meantime, nor has any effort whatever been

HOWE: DERMATITIS EXFOLIATIVA—INTERIOR VIEW.

made to insure their keeping. How long they will retain this power of self-preservation I am unable to say, but a similar specimen exfoliated by this same person five years ago, is still in my possession and shows no sign whatever of disintegration.

The appearance of the skin also varied greatly in different parts of the body. In some places it looked as if it had been blistered. In fact, it was not an uncommon occurrence to hear the sufferer declare that he was being boiled alive. This was more especially so on the hands and feet. Some areas of the epidermis were more intensely inflamed than others. It was simply a question of degree. The higher the degree the more destructive was its effect upon the tissues. As might be expected, the underlying skin also presented a variable appearance. In some places it was comparatively smooth and healthy, the inflammation not having invaded it to any great extent. In others, it was rough and mottled and suffered partial destruction, while in others it was highly reddened, completely destroyed, and underwent a secondary exfoliation. In many places numerous bleeding puncta were seen beneath the desquamated surfaces. The itching or pruritis which attended the case was something terrible. This, which was an early symptom, grew progressively worse, reaching its height between the ninth and eleventh days. There was an irresistible desire to scratch, and though his hands were well bandaged, he succeeded in inflicting many bleeding injuries with his finger nails. Excessive thirst was another persistent symptom throughout the disease. This I attributed to the inflammation, involving the mucous membrane of the mouth and throat, and to a marked impairment of the action of the salivary glands.

The nails of the hands and feet lost their lustre, died, and in time were replaced by new ones. The hair fell in quantities; it finally grew again, but not in abundance. The skin remained in a dried dessicated condition for weeks. That of the hands and feet was the last to regain its healthy appearance.

Though the man is now in an apparently healthy condition, who can tell when he will again fall a victim to this distressing disease, or who can offer a prophylaxis, which will save him from its tortures?

Case II.—E. G., aged 3 years; female; family history good, child of strong, healthy parents ; never had any severe illness prior to the invasion of this attack. First began to complain on August 25, 1899. Early symptoms were loss of appetite, nausea, headache, indisposition to play and general lassitude. The next day the child was brought to me at my office. She was pale, yet gave a temperature of 102°. She said "her head pained her" and that "she felt sick all over." Had been vomiting throughout the day. My first impression was that the child was suffering from acute indigestion. Put her on a carefully restricted diet, and directed the treament to her stomach and bowels. Did not hear anything from her until August 28th, when I was passing the house, and the parents asked me to stop to see her. At this time, I found the child extremely ill. Her temperature ran high, the pulse was very rapid, there was profound prostration, subsultus, delirium, rolling of head and every indication of the advent of some severe malady. The following day found no subsidence, but an aggravation of the alarming symptoms. The delirium and subsultus were now so pronounced as to lead me to believe the child was suffering from either typhoid or meningeal fever. Except a marked reduction in the pulse and a moderate fall in the temperature, the child remained in this condition for the next ten days. At this time, the father came to my office and said, "Doctor, my little girl is breaking out with some kind of an eruption, and I wish you would see her as quickly as you can." As soon as I could leave, I drove to his house and found the little sufferer one mass of eruption. It had made its appearance less than three hours before my visit, and now covered her entire body. Her eyes were red, face diffused and badly swollen and I was forced to change my opinion regarding the diagnosis of the case. What had so far been an extremely singular case, was rendered more so, and, as in the former case, I was at a loss what to call it. There had been no history of exposure to any of the exanthematous diseases, and yet the child presented every indication of a typical case of measles. This extensive eruption continued for two days and then gradually subsided. With its subsidence came a marked improvement of the constitutional symptoms, and the general condition of the child. My mind was beginning to be at ease, when another hasty summons informed me that my patient was again breaking out, and to all appearances, precisely as before. On reaching her bedside, I found the same to be true. The same rapid, extensive and violent dermatitis had again appeared, and the child was once more a sight to be seen. Unlike the former attack this one did not subside in a few days but persisted for weeks. Except in this and the other cases included in this paper, I have never seen any dermatitis approach it in severity. As might be expected, it wrought great destruction to the epidermis, which rolled up in great sheets, extending in some cases from the ensiform cartilage nearly to the pubes. Again, great sheets would roll up on the back as if the child had been scalded. In this way the entire epidermis desquamated, leaving a new skin in its place. But this did not escape the ravages of the disease. No sooner would the old epidermis loosen and exfoliate than the new skin would be attacked by the inflammation and destroyed.

This dreadful condition continued without abatement for nearly six weeks. During this time, the child became rapidly emaciated, the blood greatly impoverished, and the picture was a sad one to look upon. The lips, the mouth, the anterior and posterior nares were one mass of bleeding sores. Several severe attacks of epitaxsis complicated the case in its late stage, and were controlled only by the most heroic measures.

Profound anemia supervened, and ecchymosis of the eyelids was added as the little sufferer lingered. On October 7, 1899, or six weeks after the invasion of the disease, complete suppression of the urine arose.

Catheterisation of the bladder found it empty. The child gradually became comatose and died on October 9, 1899, of uremia, with interstitial nephritis. In this case, as in the former, many children were freely exposed, but none contracted it. There were children in both families and every possible exposure was offered, with negative results.

## Figures and Tables

**Figure f1:**
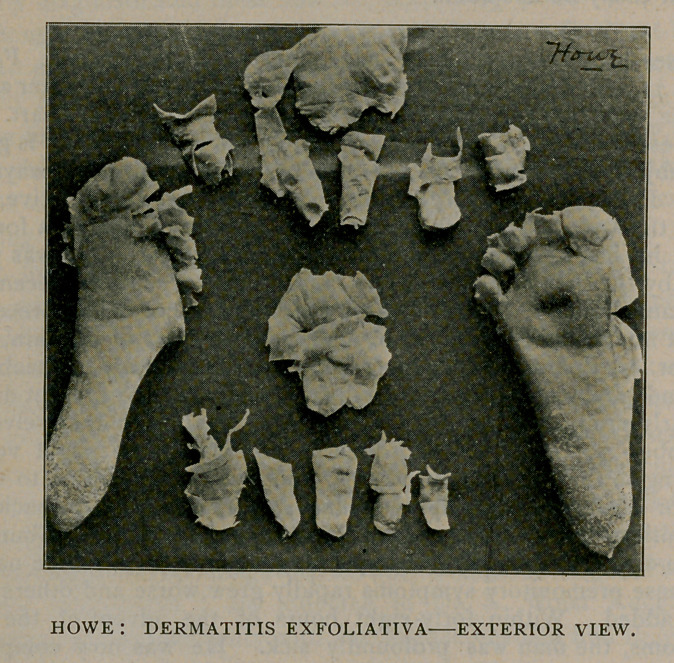


**Figure f2:**